# Neural Modularity Helps Organisms Evolve to Learn New Skills without Forgetting Old Skills

**DOI:** 10.1371/journal.pcbi.1004128

**Published:** 2015-04-02

**Authors:** Kai Olav Ellefsen, Jean-Baptiste Mouret, Jeff Clune

**Affiliations:** 1 Department of Computer and Information Science, Norwegian University of Science and Technology, Trondheim, Norway; 2 Sorbonne Université UPMC Univ Paris 06, UMR 7222, ISIR, Paris, France; 3 CNRS, UMR 7222, ISIR, Paris, France; 4 Computer Science Department, University of Wyoming, Laramie, Wyoming, United States of America; University of Vermont, UNITED STATES

## Abstract

A long-standing goal in artificial intelligence is creating agents that can learn a variety of different skills for different problems. In the artificial intelligence subfield of neural networks, a barrier to that goal is that when agents learn a new skill they typically do so by losing previously acquired skills, a problem called *catastrophic forgetting*. That occurs because, to learn the new task, neural learning algorithms change connections that encode previously acquired skills. How networks are organized critically affects their learning dynamics. In this paper, we test whether catastrophic forgetting can be reduced by evolving *modular* neural networks. Modularity intuitively should reduce learning interference between tasks by separating functionality into physically distinct modules in which learning can be selectively turned on or off. Modularity can further improve learning by having a reinforcement learning module separate from sensory processing modules, allowing learning to happen only in response to a positive or negative reward. In this paper, learning takes place via neuromodulation, which allows agents to selectively change the rate of learning for each neural connection based on environmental stimuli (e.g. to alter learning in specific locations based on the task at hand). To produce modularity, we evolve neural networks with a cost for neural connections. We show that this *connection cost technique* causes modularity, confirming a previous result, and that such sparsely connected, modular networks have higher overall performance because they learn new skills faster while retaining old skills more and because they have a separate reinforcement learning module. Our results suggest (1) that encouraging modularity in neural networks may help us overcome the long-standing barrier of networks that cannot learn new skills without forgetting old ones, and (2) that one benefit of the modularity ubiquitous in the brains of natural animals might be to alleviate the problem of catastrophic forgetting.

## Author Summary

A long-standing goal in artificial intelligence (AI) is creating computational brain models (neural networks) that learn what to do in new situations. An obstacle is that agents typically learn new skills only by losing previously acquired skills. Here we test whether such forgetting is reduced by evolving modular neural networks, meaning networks with many distinct subgroups of neurons. Modularity intuitively should help because learning can be selectively turned on only in the module learning the new task. We confirm this hypothesis: modular networks have higher overall performance because they learn new skills faster while retaining old skills more. Our results suggest that one benefit of modularity in natural animal brains may be allowing learning without forgetting.

## Introduction

A long-standing scientific challenge is to create agents that can *learn*, meaning they can adapt to novel situations and environments within their lifetime. The world is too complex, dynamic, and unpredictable to program all beneficial strategies ahead of time, which is why robots, like natural animals, need to be able to continuously learn new skills on the fly.

Having robots learn a large set of skills, however, has been an elusive challenge because they need to learn new skills without forgetting previously acquired skills [1–3]. Such forgetting is especially problematic in fields that attempt to create artificial intelligence in brain models called artificial neural networks [1, 4, 5]. To learn new skills, neural network learning algorithms change the *weights* of neural connections [6–8], but old skills are lost because the weights that encoded old skills are changed to improve performance on new tasks. This problem is known as *catastrophic forgetting* [9, 10] to emphasize that it contrasts with biological animals (including humans), where there is *gradual* forgetting of old skills as new skills are learned [11]. While robots and artificially intelligent software agents have the potential to significantly help society [12–14], their benefits will be extremely limited until we can solve the problem of catastrophic forgetting [1, 15]. To advance our goal of producing sophisticated, functional artificial intelligence in neural networks and make progress in our long-term quest to create general artificial intelligence with them, we need to develop algorithms that can learn how to handle more than a few different problems. Additionally, the difference between computational brain models and natural brains with respect to catastrophic forgetting limits the usefulness of such models as tools to study neurological pathologies [16].

**Fig 1:**
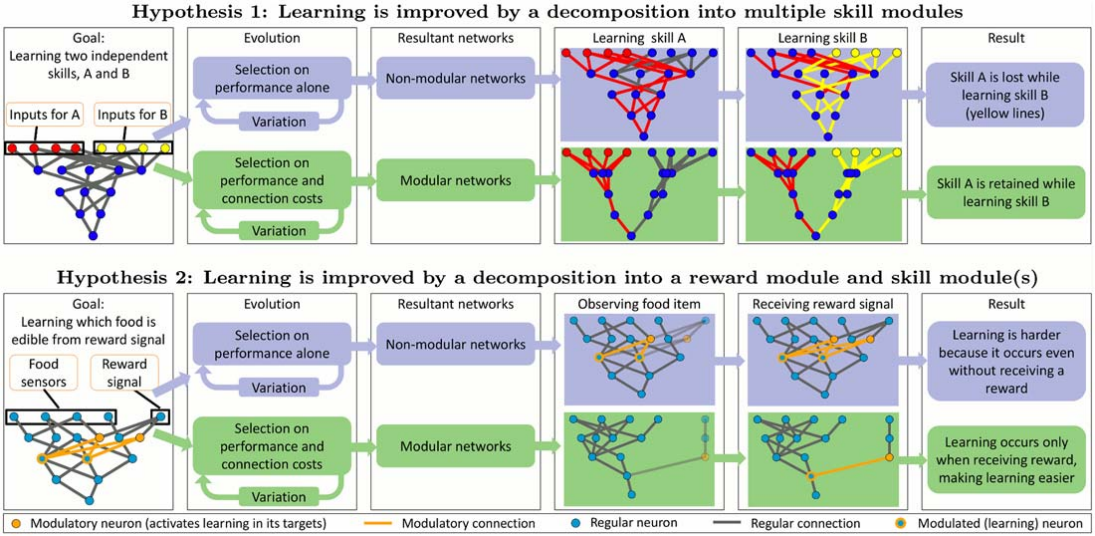
Two hypotheses for how neural modularity can improve learning. Hypothesis 1: Evolving non-modular networks leads to the forgetting of old skills as new skills are learned. Evolving networks with a pressure to minimize connection costs leads to modular solutions that can retain old skills as new skills are learned. Hypothesis 2: Evolving modular networks makes reward-based learning easier, because it allows a clear separation of reward signals and learned skills. We present evidence for both hypotheses in this paper.

In this paper, we investigate the hypothesis that modularity, which is widespread in biological neural networks [17–21], helps reduce catastrophic forgetting in artificial neural networks. Modular networks are those that have many clusters (modules) of highly connected neurons that are only sparsely connected to neurons in other modules [19, 22, 23]. The intuition behind this hypothesis is that modularity could allow learning new skills without forgetting old skills because learning can be selectively turned on only in modules learning a new task (Fig. 1, top). Selective regulation of learning occurs in natural brains via *neuromodulation* [24], and we incorporate an abstraction of it in our model [25]. We also investigate a second hypothesis: that modularity can improve skill learning by separating networks into a *skill module* and a *reward module*, resulting in more precise control of learning (Fig. 1, bottom).

To evolve *modular* networks, we add another natural phenomenon: costs for neural connections. In nature, there are many costs associated with neural connections (e.g. building them, maintaining them, and housing them) [26–28] and it was recently demonstrated that incorporating a cost for such connections encourages the evolution of modularity in networks [23]. Our results support the hypothesis that modularity does mitigate catastrophic forgetting: modular networks have higher overall performance because they learn new skills faster while retaining old skills more. Additional research into this area, including investigating the generality of our results, will catalyze research on creating artificial intelligence, improve models of neural learning, and shed light on whether one benefit of modularity in natural animal brains is an improved ability to learn without forgetting.

### Background

#### Catastrophic forgetting

Catastrophic forgetting (also called catastrophic interference) has been identified as a problem for artificial neural networks (ANNs) for over two decades: When learning multiple tasks in a sequence, previous skills are forgotten rapidly as new information is learned [9, 10]. The problem occurs because learning algorithms only focus on solving the current problem and change any connections that will help solve that problem, even if those connections encoded skills appropriate to previously encountered problems [9].

Many attempts have been made to mitigate catastrophic forgetting. *Novelty vectors* modify the backpropagation learning algorithm [7] to limit the number of connections that are changed in the network based on how novel, or unexpected, the input pattern is [29]. This technique is only applicable for auto-encoder networks (networks whose target output is identical to their input), thus limiting its value as a general solution to catastrophic forgetting [1]. *Orthogonalization* techniques mitigate interference between tasks by reducing their representational overlap in input neurons (via manually designed preprocessing) and by encouraging sparse hidden-neuron activations [30–32]. *Interleaved learning* avoids catastrophic forgetting by training on both old and new data when learning [10], although this method cannot scale and does not work for realistic environments because in the real world not all challenges are faced concurrently [33, 34]. This problem with interleaved learning can be reduced with *pseudo rehearsal*, wherein input-output associations from old tasks are remembered and rehearsed [34]. However, scaling remains an issue with pseudo rehearsal because such associations still must be stored and choosing which associations to store is an unsolved problem [15]. These techniques are all engineered approaches to reducing the problem of catastrophic forgetting and are not proposed as methods by which natural evolution solved the problem of catastrophic forgetting [1, 10, 29–32, 34].

*Dual-net architectures*, on the other hand, present a biologically plausible [35] mechanism for limiting catastrophic forgetting [33, 36]. The technique, inspired by theories on how human brains separate and subsequently integrate old and new knowledge, partitions early processing and long-term storage into different subnetworks. Similar to interleaved learning techniques, dual-net architectures enable both new knowledge and input history (in the form of current network state) to affect learning.

Although these methods have been suggested for reducing catastrophic forgetting, many questions remain about how animals avoid this problem [1] and which mechanisms can help avoid it in neural networks [1, 15]. In this paper, we study a new hypothesis, which is that *modularity* can help avoid catastrophic forgetting. Unlike the techniques mentioned so far, our solution does not require human design, but is automatically generated by evolution. Evolving our solution under biologically realistic constraints has the added benefit of suggesting how such a mechanism may have originated in nature.

#### Evolving neural networks that learn

One method for setting the connection weights of neural networks is to evolve them, meaning that an *evolutionary algorithm* specifies each weight, and the weight does not change within an organism’s “lifetime” [5, 37–39]. Evolutionary algorithms abstract Darwinian evolution: in each generation a population of “organisms” is subjected to selection (for high performance) and then mutation (and possibly crossover) [5, 38]. These algorithms have shown impressive performance—often outperforming human engineers [40, 41]—on a range of tasks, such as measuring properties in quantum physics [12], dynamic rocket guidance [42], and robot locomotion [43, 44].

Another approach to determining the weights of neural networks is to initialize them randomly and then allow them to change via a learning algorithm [5, 7, 45]. Some learning algorithms, such as backpropagation [6, 7], require a correct output (e.g. action) for each input. Other learning algorithms are considered more biologically plausible in that they involve only information local to each neuron (e.g. Hebb’s rule [45]) or infrequent reward signals [8, 46, 47].

Evolution and learning can be combined, wherein evolution creates an initial neural network and then a learning algorithm modifies its connections within the lifetime of the organism [5, 37, 47–49]. Compared to behaviors defined solely by evolution, evolving agents that learn leads to better solutions in fewer generations [48, 50, 51], improved adaptability to changing environments [48, 49], and enables evolving solutions for larger neural networks [48]. Computational studies of evolving agents that learn have also shed light on open biological questions regarding the interactions between evolution and learning [50, 52, 53].

The idea of using evolutionary computation to reduce catastrophic forgetting has not been widely explored. In one relevant paper, evolution optimized certain parameters of a neural network to mitigate catastrophic forgetting [15]. Such parameters included the number of hidden (internal) neurons, learning rates, patterns of connectivity, initial weights, and output error tolerances. That paper did show that there is a potential for evolution to generate a stronger resistance to catastrophic forgetting, but did not investigate the role of modularity in helping produce such a resistance.

#### Neuromodulatory learning in neural networks

Evolutionary experiments on artificial neural networks typically model only the classic excitatory and inhibitory actions of neurons in the brain [5]. In addition to these processes, biological brains employ a number of different *neuromodulators*, which are chemical signals that can locally modify learning [24, 54, 55]. By allowing evolution to design neuromodulatory dynamics, learning rates for particular synapses can be upregulated and downregulated in response to certain inputs from the environment. These additional degrees of freedom greatly increase the possible complexity of reward-based learning strategies. This type of plasticity-controlling neuromodulation has been successfully applied when evolving neural networks that solve reinforcement learning problems [25, 46], and a comparison found that evolution was able to solve more complex tasks with neuromodulated Hebbian learning than with Hebbian learning alone [25]. Our experiments include this form of neuromodulation (Methods).

#### Evolved modularity in neural networks

Modularity is ubiquitous in biological networks, including neural networks, genetic regulatory networks, and protein interaction networks [17–21]. Why modularity evolved in such networks has been a long-standing area of research [18–20, 56–59]. Researchers have also long studied how to encourage the evolution of modularity in artificial neural networks, usually by creating the conditions that are thought to promote modularity in natural evolution [19, 57–61]. Several different hypotheses have been suggested for the evolutionary origins of modularity.

A leading hypothesis has been that modularity emerges when evolution occurs in rapidly changing environments that have common subproblems, but different overall problems [57]. These environments are said to have *modularly varying goals*. While such environments can promote modularity [57], the effect only appears for certain frequencies of environmental change [23] and can fail to appear with different types of networks [58, 60, 61]. Moreover, it is unclear how many natural environments change *modularly* and how to design training problems for artificial neural networks that have modularly varying goals. Other experiments have shown that modularity may arise from gene duplication and differentiation [19], or that it may evolve to make networks more robust to noise in the genotype-phenotype mapping [58] or to reduce interference between network activity patterns [59].

Recently, a different cause of module evolution was documented: that modularity evolves when there are costs for connections in networks [23]. This explanation for the evolutionary origins of modularity is biologically plausible because biological networks have connection costs (e.g. to build connections, maintain them, and house them) and there is evidence that natural selection optimally arranges neurons to minimize these connection costs [26, 27]. Moreover, the modularity-inducing effects of adding a connection cost were shown to occur in a wide range of environments, suggesting that adding a selection pressure to reduce connection costs is a robust, general way to encourage modularity [23]. We apply this technique in our paper because of its efficacy and because it may be a main reason that modularity evolves in natural networks.

### Experimental Setup

To test our hypotheses, we set up an environment in which there is a potential for catastrophic forgetting and where individuals able to avoid this forgetting receive a higher evolutionary *fitness*, meaning they are more likely to reproduce. The environment is an abstraction of a world in which an organism performs a daily routine of trying to eat nutritious food while avoiding eating poisonous food. Every day the organism observes every food item one time: half of the food items are nutritious and half are poisonous. To achieve maximum fitness, the individual needs to eat all the nutritious items and avoid eating the poisonous ones. After a number of days, the season changes abruptly from a summer season to a winter season. In the new season, there is a new set of food sources, half of them nutritious and half poisonous, and the organism has to learn which is which. After this winter season, the environment changes back to the summer season and the food items and their nutritious/poisonous statuses are the same as in the previous summer. The environment switches back and forth between these two seasons multiple times in the organism’s lifetime. Individuals that remember each season’s food associations perform better by avoiding poisonous items without having to try them first.

**Fig 2:**
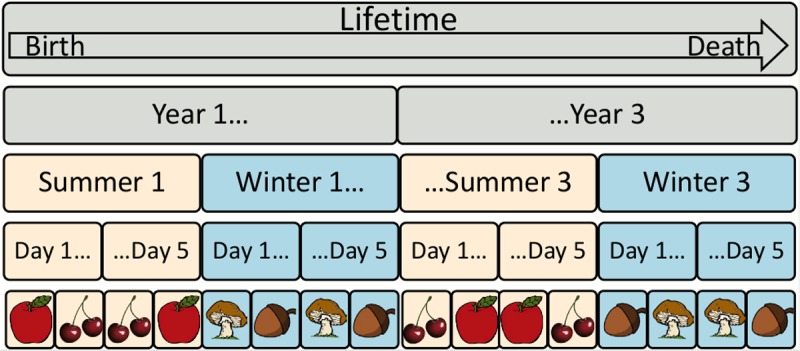
The environment for one individual’s lifetime. A lifetime lasts 3 years. Each year has 2 seasons: winter and summer. Each season consists of 5 days. In each day, each individual sees all food items available in that season (only two are shown) in a random order.

**Fig 3:**
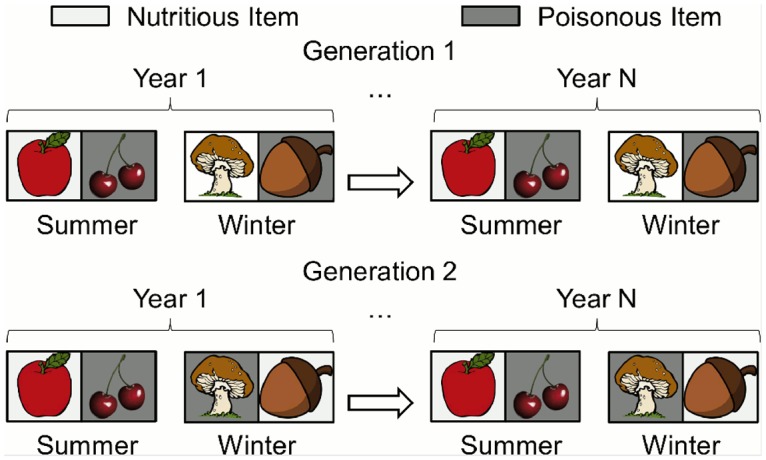
Randomizing food associations between generations. To ensure that agents learn associations within their lifetimes instead of genetically hardcoding associations, whether each food item is nutritious or poisonous is randomized each generation. There are four food items per season (two are depicted).

We consider each pair of a summer and winter season a *year*. Every season lasts for five *days*, and in each day an individual encounters all four food items for that season in a random order. A *lifetime* is three years (Fig. 2). To ensure that individuals must *learn* associations within their lifetimes instead of having genetically hardcoded associations [47, 62], in each lifetime two food items are randomly assigned as nutritious and the other two food items are assigned as poisonous (Fig. 3). To select for *general* learners rather than individuals that by chance do well in a specific environment, performance is averaged over four random environments (lifetimes) for each individual during evolution, and over 80 random environments (lifetimes) when assessing the performance of final, end-of-experiment individuals (Methods).

This environment selects for agents that can avoid forgetting old information as they learn new, unrelated information. For instance, if an agent is able to avoid forgetting the summer associations during the winter season, it will immediately perform well when summer returns, thus outcompeting agents that have to relearn summer associations. Agents that forget, especially catastrophically, are therefore at a selective disadvantage.

Our main results were found to be robust to variations in several of our experimental parameters, including changes to the number of years in the organism’s lifetime, the number of different seasons per year, the number of different edible items, and different representations of the inputs (the presence of items being represented either by a single input or distributed across all inputs for a season). We also observed that our results are robust to lengthening the number of days per season: networks in the experimental treatment (called “P&CC” for reasons described below) significantly outperform the networks in the control (“PA”) treatment (*p* < 0.05) even when doubling or quadrupling the number of days per season, although the size of the difference diminished in longer seasons.

#### Neural network model

The model of the organism’s brain is a neural network with 10 input neurons (Supp. S1 Fig). From left to right, inputs 1-4 and 5-8 encode which summer and winter food item is present, respectively. During summer, the winter inputs are never active and vice versa. Catastrophic forgetting may appear in these networks because a non-modular neural network is likely to use the same hidden neurons for both seasons (Fig. 1, top). We segmented the summer and winter items into separate input neurons to abstract a neural network responsible for an intermediate phase of cognition, where early visual processing and object recognition have already occurred, but before decisions have been made about what to do in response to the recognized visual stimuli. Such disentangled representations of objects have been identified in animal brains [63] and are common at intermediate layers of neural network models [64]. The final two inputs are for reinforcement learning: inputs 9 and 10 are reward and punishment signals that fire when a nutritious or poisonous food item is eaten, respectively. The network has a single output that determines if the agent will eat (*output* > 0) or ignore (*output* < = 0) the presented food item.

Associations can be learned by properly connecting reward signals through neuromodulatory neurons to non-modulatory neurons that determine which actions to take in response to food items (Methods). Evolution determines the neural wiring that produces learning dynamics, as described next.

#### Evolutionary algorithm

Evolution begins with a randomly generated population of neural networks. The *performance* of each network is evaluated as described above. More fit networks tend to have more offspring, with fitness being determined differently in each treatment, as explained below. Offspring are generated by copying a parent genome and mutating it by adding or removing connections, changing the strength of connections, and switching neurons from being modulatory to non-modulatory or vice versa. The process repeats for 20,000 generations.

To evolve *modular* neural networks, we followed a recently demonstrated procedure where modularity evolves as a byproduct of a selection pressure to reduce neural connectivity [23]. We compared a treatment where the fitness of individuals was based on performance alone (PA) to one based on both maximizing performance and minimizing connection costs (P&CC). Specifically, evolution proceeds according to a multi-objective evolutionary algorithm with one (PA) or two (P&CC) primary objectives. A network’s connection cost equals its number of connections, following [23]. More details on the evolutionary algorithm can be found in Methods.

## Results

### A Connection Cost Increases Performance and Modularity

**Fig 4:**
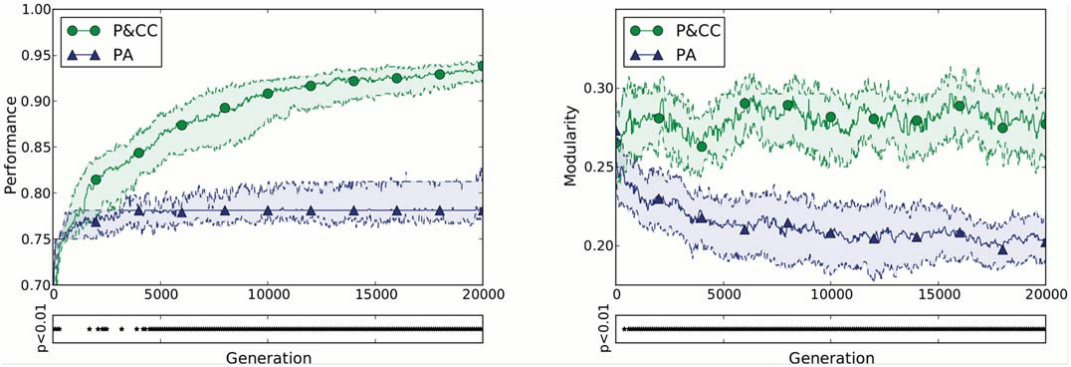
The addition of a cost for network connections, which is present only in the P&CC treatment, significantly increases performance and modularity. Modularity is measured via a widely used approximation of the standard *Q* modularity score [23, 57, 65, 67] (Methods). For each treatment, the median from 100 independent evolution experiments is shown ± 95% bootstrapped confidence intervals of the median (Methods). Asterisks below each plot indicate statistically significant differences at *p* < 0.01 according to the Mann-Whitney U test, which is the default statistical test throughout this paper unless otherwise specified.

**Fig 5:**
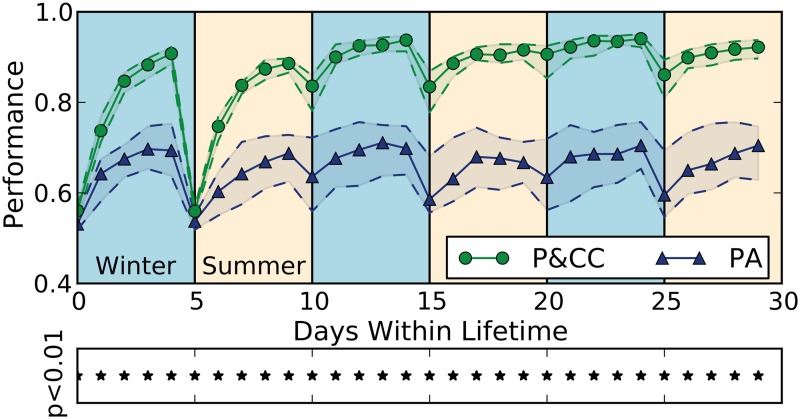
Performance each day for evolved agents from both treatments. Plotted is median performance per day (± 95% bootstrapped confidence intervals of the median) measured across 100 organisms (the highest-performing organism from each experiment per treatment) tested in 80 new environments (lifetimes) with random associations (Methods). P&CC networks significantly outperform PA networks on every day (asterisks). Eating no items or all items produces a score of 0.5; eating all and only nutritious food items achieves the maximum score of 1.0.

The addition of a cost for connections (the P&CC treatment) leads to a rapid, sustained, and statistically significant fitness advantage versus not having a connection cost (the PA treatment) (Fig. 4). In addition to overall performance across generations, we looked at the day-to-day performance of final, evolved individuals (Fig. 5). P&CC networks learn associations faster in their first summer and winter, and maintain higher performance over multiple years (pairs of seasons).

**Fig 6:**
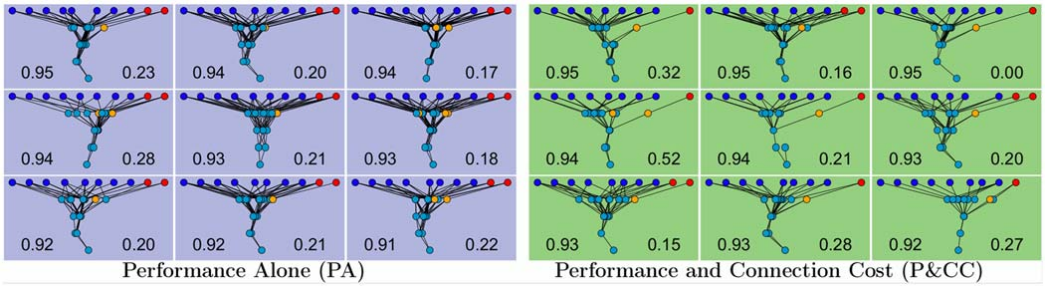
PA networks are visually non-modular whereas P&CC networks tend to create a separate module for learning (red and orange neurons), as hypothesized in Fig. 1 (bottom). Dark blue nodes are inputs that encode which type of food has been encountered. Light blue nodes indicate internal, non-modulatory neurons. Red nodes are reward or punishment inputs that indicate if a nutritious or poisonous item has been eaten. Orange neurons are neuromodulatory neurons that regulate learning. P&CC networks tend to separate the reward/punishment inputs and neuromodulatory neurons into a separate module that applies learning to downstream neurons that determine which actions to take. For each treatment, the highest-performing network from each of the nine highest-performing evolution experiments are shown (all are shown in the Supporting Information). In each panel, the left number reports performance and the right number reports modularity. We follow the convention from [23] of placing nodes in the way that minimizes the total connection length.

The presence of a connection cost also significantly increases network *modularity* (Fig. 4), confirming the finding of Clune et al. [23] in this different context of networks with within-life learning. Networks evolved in the P&CC treatment tend to create a separate reinforcement learning module that contains the reward and punishment inputs and most or all neuromodulatory neurons (Fig. 6). One of our hypotheses (Fig. 1, bottom) suggested that such a separation could improve the efficiency of learning, by regulating learning (via neuromodulatory neurons) in response to whether the network performed a correct or incorrect action, and applying that learning to downstream neurons that determine which action should be taken in response to input stimuli.

To quantify whether learning is separated into its own module, we adopted a technique from [23], which splits a network into the most modular decomposition according to the modularity *Q* score [65]. We then measured the frequency with which the reinforcement inputs (reward/punishment signals) were placed into a different module from the remaining food-item inputs. This measure reveals that P&CC networks have a separate module for learning in 31% of evolutionary trials, whereas only 4% of the PA trials do, which is a significant difference (*p* = 2.71 × 10^−7^), in agreement with our hypothesis (Fig. 1, bottom). Analyses also reveal that the networks from both treatments that have a separate module for learning perform significantly better than networks without this decomposition (median performance of modular networks in 80 randomly generated environments (Methods): 0.87 [95% CI: 0.83, 0.88] vs. non-modular networks: 0.80 [0.71, 0.84], *p* = 0.02). Even though only 31% of the P&CC networks are deemed modular in this particular way, the remaining P&CC networks are still significantly more modular on average than PA networks (median Q scores are 0.25 [0.23, 0.28] and 0.2 [0.19, 0.22] respectively, *p* = 4.37 × 10^−6^), suggesting additional ways in which modularity improves the performance of P&CC networks.

**Fig 7:**
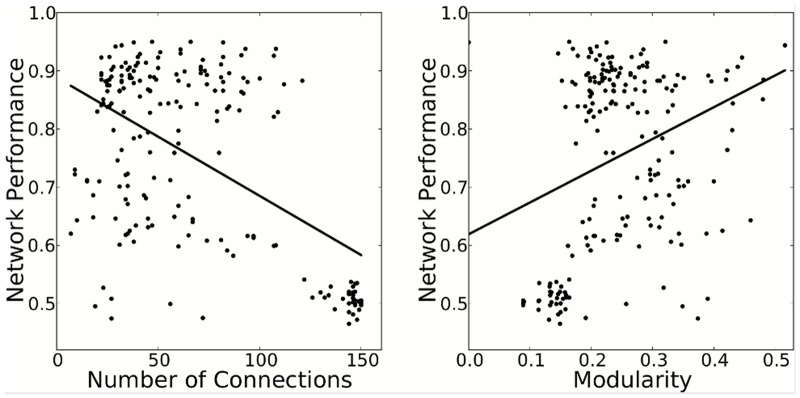
Performance is correlated with sparsity and modularity. Black dots represent the highest-performing network from each of the 100 experiments from both the PA and P&CC treatments. Both the sparsity (*p* = 1.08 × 10^−16^) and modularity (*p* = 1.19 × 10^−5^) of networks significantly correlates with their performance. Performance was measured in 80 randomly generated environments (Methods). Significance was calculated by a *t*-test of the hypothesis that the correlation is zero. Notice that many of the lowest-performing networks are close to the maximum of 150 connections.

After observing that a connection cost significantly improves performance and modularity, we analyzed whether this increased performance can be explained by the increased modularity, or whether it may better correlate with network *sparsity*, since P&CC networks also have fewer connections (P&CC median number of connections is 35.5 [95% CI: 31.0, 40.0] vs. PA 82.0 [74.0, 97.1], *p* = 7.97 × 10^−19^). Both sparsity and modularity are correlated with the performance of networks (Fig. 7). Sparsity also correlates with modularity (*p* = 5.15 × 10^−40^ as calculated by a *t*-test of the hypothesis that the correlation is zero), as previously shown [23, 66]. Our interpretation of the data is that the pressure for both functionality and sparsity causes modularity, which in turn helps evolve learners that are more resistant to catastrophic forgetting. However, it cannot be ruled out that sparsity itself mitigates catastrophic forgetting [1], or that the *general* learning abilities of the network have been improved due to the separation into a skill module and a learning module. Either way, the data support our hypothesis that a connection cost promotes the evolution of sparsity, modularity, and increased performance on learning tasks.

### Modular P&CC Networks Learn More and Forget Less

We next investigated whether the improved performance of P&CC individuals is because they forget less. Measuring the percent of information a network retains can be misleading, because networks that never learn anything are reported as never forgetting anything. In many PA experiments, networks did not learn in one or both seasons, which looks like perfect *retention*, but for the wrong reason: they do not forget anything because they never knew anything to begin with. To prevent such pathological, non-learning networks from clouding this analysis, we compared only the 50 highest-performing experiments from each treatment, instead of all 100 experiments. For both treatments, we then measured retention and forgetting in the highest-performing network from each of these 50 experiments.

To illuminate how old associations are forgotten and new ones are formed, we performed an experiment from studies of association forgetting in humans [11]: already evolved individuals learned one task and then began training on a new task, during which we measured how their performance on the original task degraded. Specifically, we allowed individuals to learn for 50 winter days—to allow even poor learners time to learn the winter associations—before exposing them to 20 summer days, during which we measured how rapidly they forgot winter associations and learned summer associations (Methods). Notice that individuals were evolved in seasons lasting only 5 days, but we measure learning and forgetting for 20 days in this analysis to study the longer-term consequences of the evolved learning architectures. Thus, the key result relevant to catastrophic forgetting is what occurs during the first five days. We included the remaining 15 days to show that the differences in performance persist if the seasons are extended.

**Fig 8:**
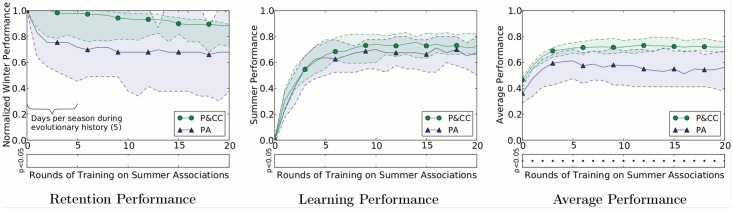
Comparing the retention and forgetting of networks from the two treatments. P&CC networks, which are more modular, are better at *retaining* associations learned on a previous task (winter associations) while learning a new task (summer associations), better at *learning* new (summer) associations, and significantly better when measuring performance on *both* the associations for the original task (winter) and the new task (summer). Note that networks were evolved with five days per season, so the results during those first five days are the most informative regarding the evolutionary mitigation of catastrophic forgetting: we show additional days to reveal longer-term consequences of the evolved architectures. Solid lines show median performance and shaded areas indicate 95% bootstrapped confidence intervals of the median. The retention scores (left panel) are normalized relative to the original performance before training on the new task (an unnormalized version is provided as Supp. S6 Fig). During all performance measurements, learning was disabled to prevent such measurements from changing an individual’s known associations (Methods).

P&CC networks retain higher performance on the original task when learning a new task (Fig. 8, left). They also learn the new task better (Fig. 8, center). The combined effect significantly improves performance (Fig. 8, right), meaning P&CC networks are significantly better at learning associations in a new season while retaining associations from a previous one.

To further understand whether the increased performance of the P&CC individuals is because they learn more, retain more, or both, we counted the number of retained and learned associations for individuals in 80 randomly generated environments (lifetimes). If we regard performance in each season as a *skill*, this experiment measures whether the individuals can retain a previously-learned skill (perfect summer performance) after learning a new skill (perfect winter performance). We tested the knowledge of the individuals in the following way: at the end of each season, we counted the number of sets of associations (summer or winter) that individuals knew perfectly, which required them knowing the correct response for each food item in that season. We formulated four metrics that quantify how well individuals knew and retained associations.

**Fig 9:**
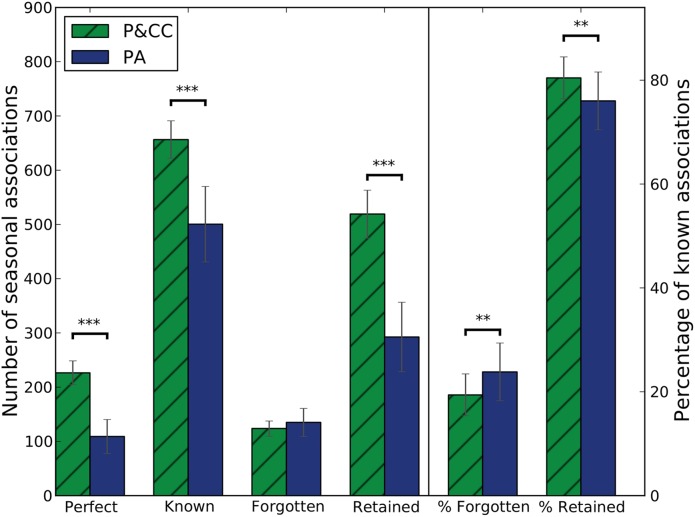
P&CC networks significantly outperform PA networks in both learning and retention. P&CC individuals learn significantly more associations, whether counting only when the associations for both seasons are known (“Perfect” knowledge) or separately counting knowledge of either season’s association (total “Known”). P&CC networks also forget fewer associations, defined as associations known in one season and then forgotten in the next, which is significant when looking at the percent of known associations forgotten (“% Forgotten”). P&CC networks also retain significantly more associations, meaning they did not forget one season’s association when learning the next season’s association. See text for more information about the “Perfect”, “Known”, “Forgotten,” and “Retained” metrics. During all performance measurements, learning was disabled to prevent such measurements from changing an individual’s known associations (Methods). Bars show median performance, whiskers show the 95% bootstrapped confidence interval of the median. Two asterisks indicate *p* < 0.01, three asterisks indicate *p* < 0.001.

The first metric (“*Perfect*”) measures the number of seasons an individual knew *both* sets of associations (summer and winter). Doing well on this metric indicates reduced catastrophic forgetting because it requires retaining an old skill even after a new one is learned. P&CC individuals learned significantly more Perfect associations (Fig. 9, Perfect).

The second metric (“*Known*”) is the sum of the number of seasons that summer associations were known and the number of seasons that winter associations were known. In other words, it counts knowing either season in a year and doubly counts knowing both. P&CC individuals learned significantly more of these Known associations (Fig. 9, Known).

The third metric counts the number of seasons in which an association was “*Forgotten*”, meaning an association was completely known in one season, but was not in the following season. There is no significant difference between treatments on this metric when measured in absolute numbers (Fig. 9, Forgotten). However, measured as a percentage of *Known* items, P&CC individuals forgot significantly fewer associations (Fig. 9, % Forgotten). The modular P&CC networks thus learned more and forgot less—leading to a significantly lower *percentage* of forgotten associations.

The final metric counts the number of seasons in which an association was “*Retained*”, meaning an association was completely known in one season and the following season. P&CC individuals retained significantly more than PA individuals, both in absolute numbers (Fig. 9, Retained) and as a percentage of the total number of known items (Fig. 9, % Retained).

In each season, an agent can know two associations (summer and winter), leading to a maximum score of 6 × 80 × 2 = 960 for the *known* metric (6 seasons per lifetime (Fig. 2), 80 random environments). The agent can retain or forget two associations each season except the first, making the maximum score for these metrics 5 × 80 × 2 = 800. However, the agent can only score one perfect association (meaning both summer and winter is known) each season, leading to a maximum score of 6 × 80 = 480 for that metric.

In summary, this analysis reveals that a connection cost caused evolution to find individuals that are better at gaining new knowledge without forgetting old knowledge. In other words, adding a connection cost mitigated catastrophic forgetting. That, in turn, enabled an increase in the total number of associations P&CC individuals learned in their lifetimes.

### Removing the Ability of Evolution to Improve Retention

To further test whether the improved performance in the P&CC treatment results from it mitigating catastrophic forgetting, we conducted experiments in a regime where retaining skills between tasks is impossible. Under such a regime, if the P&CC treatment does not outperform the PA treatment, that is evidence for our hypothesis that the ability of P&CC networks to outperform PA networks in the normal regime is because P&CC networks retain previously learned skills more when learning new skills.

**Fig 10:**
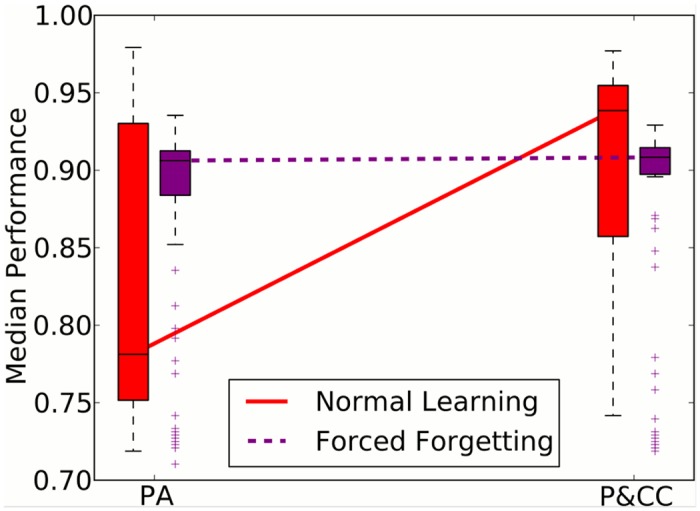
Forcing individuals to forget what they have learned in the past eliminates the performance benefits of adding a connection cost. With forced forgetting, P&CC does not significantly outperform PA: P&CC 0.91 [95% CI: 0.91, 0.91] vs. PA 0.91 [0.90, 0.91], *p* > 0.05. In the default treatment where remembering is possible, P&CC significantly outperforms PA: P&CC 0.94 [0.92, 0.94] vs. PA 0.78 [0.78, 0.81], *p* = 8.08 × 10^−6^.

To create a regime similar to the original problem, but without the potential to improve performance by minimizing catastrophic forgetting, we forced individuals to forget everything they learned at the end of every season. This *forced forgetting* was implemented by resetting all neuromodulated weights in the network to random values between each season change. The experimental setup was otherwise identical to the main experiment. In this treatment, evolution cannot evolve individuals to handle forgetting better, and can focus only on evolving good learning abilities for each season. With forced forgetting, the P&CC treatment no longer significantly outperforms the PA treatment (Fig. 10).

This result indicates that the connection cost specifically helps evolution in optimizing the parts of learning related to resistance against forgetting old associations while learning new ones.

Interestingly, without the connection cost (the PA treatment), forced forgetting significantly improves performance (Fig. 10, *p* = 2.5 × 10^−5^ via bootstrap sampling with randomization [68]). Forcing forgetting likely removes some of the interference between learning the two separate tasks. With the connection cost, however, forced forgetting leads to worse results, indicating that the modular networks in the P&CC treatment have found solutions that benefit from remembering what they have learned in the past, and thus are worse off when not allowed to remember that information.

### The Importance of Neuromodulation

We hypothesized that a key factor that causes modularity to help minimize catastrophic forgetting is *neuromodulation*, which is the ability for learning to be selectively turned on and off in specific neural connections in specific situations. To test whether neuromodulation is essential to evolving a resistance to forgetting in our experiments, we evolved neural networks *with* and *without* neuromodulation. When we evolve without neuromodulation, the Hebbian learning dynamics of each connection are constant throughout the lifetime of the organism: this is accomplished by disallowing neuromodulatory neurons from being included in the networks (Methods).

**Fig 11:**
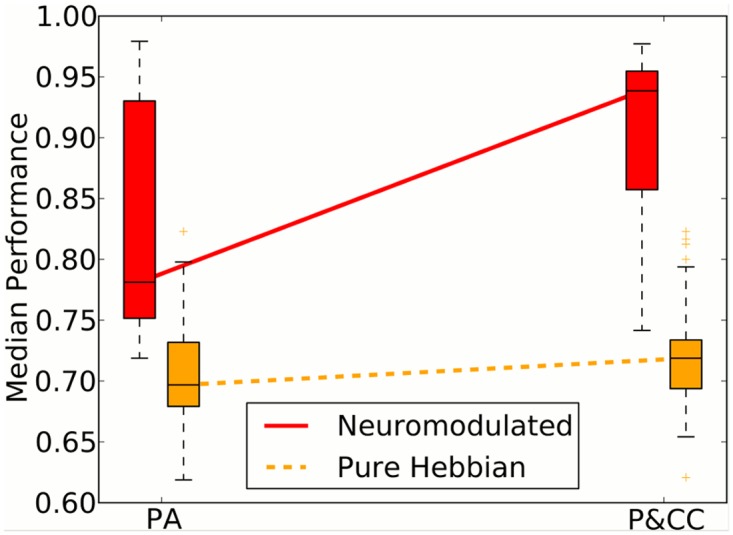
The effect of neuromodulation and connection costs when evolving solutions for catastrophic forgetting. Connection costs and neuromodulatory dynamics interact to evolve forgetting-resistant solutions. Without neuromodulation, neither treatment performs well, suggesting that neuromodulation is a prerequisite for solving these types of problems, a result that is consistent with previous research showing that neuromodulation is required to solve challenging learning tasks [25]. However, even in the non-neuromodulatory (pure Hebbian) experiments, P&CC is more modular (0.33 [95% CI: 0.33, 0.33] vs PA 0.26 [0.22, 0.31], *p* = 1.16 × 10^−12^) and performs significantly better (0.72 [95% CI: 0.71, 0.72] vs. PA 0.70 [0.69, 0.71], *p* = 0.003). That said, because both treatments perform poorly without neuromodulation, and because natural animal brains contain neuromodulated learning [28], it is most interesting to see the additional impact of modularity against the backdrop of neuromodulation. Against that backdrop, neural modularity improves performance to a much larger degree (P&CC 0.94 [0.92, 0.94] vs. PA 0.78 [0.78, 0.81], *p* = 8.08 × 10^−6^), in part by reducing catastrophic forgetting (see text).

Comparing the performance of networks evolved with and without neuromodulation demonstrates that with purely Hebbian learning (i.e. without neuromodulation) evolution never produces a network that performs even moderately well (Fig. 11). This finding is in line with previous work demonstrating that neuromodulation allows evolution to solve more complex reinforcement learning problems than purely Hebbian learning [25]. While the non-modulatory P&CC networks perform slightly better than non-modulatory PA networks, the differences, while significant (P&CC performance 0.72 [95% CI: 0.71, 0.72] vs. PA 0.70 [0.69, 0.71], *p* = 0.003), are small. Because networks in neither treatment learn much, studying whether they suffer from catastrophic forgetting is uninformative. These results reveal that neuromodulation is essential to perform well in these environments, and its presence is effectively a prerequisite for testing the hypothesis that modularity mitigates catastrophic forgetting. Moreover, neuromodulation is ubiquitous in animal brains, justifying its inclusion in our default model. One can think of neuromodulation, like the presence of neurons, as a necessary, but not sufficient, ingredient for learning without forgetting. Including it in the experimental backdrop allows us to isolate whether modularity further improves learning and helps mitigate catastrophic forgetting.

## Discussion

In the experiments we performed, we found evidence that adding a connection cost when evolving neural networks significantly increases modularity and the ability of networks to learn new skills while retaining previously learned skills. The resultant networks have a separate learning module and exhibit significantly higher performance, learning, and retention. We further found three lines of evidence that modularity improves performance and helps prevent catastrophic forgetting: (1) networks with a separate learning module performed significantly better, (2) modularity and performance are significantly correlated, and (3) the performance increase disappeared when the ability to retain skills was artificially eliminated. These findings support the idea that neural modularity can improve learning performance both for tasks with the potential for *catastrophic forgetting*, by reducing the overlap in how separate skills are stored (Fig. 1, top), and *in general*, by modularly separating learned skills from reward signals (Fig. 1, bottom).

We also found evidence supporting the hypothesis that the ability to selectively regulate per-connection learning in specific situations, called neuromodulation, is critical for the benefits of a connection cost to be realized. In the presence of neuromodulatory learning dynamics, which occur in the brains of natural animals [24, 54], a connection cost could thus significantly mitigate catastrophic forgetting. This work thus provides a new candidate technique for improving learning and reducing catastrophic forgetting, which is essential for advancing our goal of making sophisticated robots and intelligent software based on neural networks. It also suggests that one benefit of the modularity ubiquitous in natural networks may be improved learning via reduced catastrophic forgetting.

While we found these results hold in the experiments we conducted, much work remains to be done on the interesting question of how catastrophic forgetting is avoided in animal brains. Future work in different types of problems and experimental setups are needed to confirm or deny the hypotheses suggested in this paper. Specific studies that can investigate the generality of our hypothesis include studying whether the connection cost technique still reduces interference when inputs cannot be as easily disentangled (for instance, if certain inputs are shared between several skills), investigating the effect of more complex learning tasks that may not be learned at all if the agent forgets between training episodes, and further exploring the effect of experimental parameters, such as the length of training episodes, number of tasks, and different neural network sizes and architectures.

Additionally, while we focused primarily on *evolution* specifying modular architectures, those architectures could also emerge via intra-life learning rules that lead to modular neural architectures. In fact, there may have been evolutionary pressure to create learning dynamics that result in neural modularity: whether such “modular plasticity” rules exist, how they mechanistically cause modularity, and the role of evolution in producing them, is a ripe area for future study. More generally, exploring the degree to which evolution encodes learning rules that lead to modular architectures, as opposed to hard coding modular architectures, is an interesting area for future research.

The experiments in this paper are meant to invigorate the conversation about how evolution and learning produce brains that avoid catastrophic forgetting. While the results of these experiments shed light on that question, the importance, magnitude, and complexity of the question will yield fascinating research for decades, if not centuries, to come.

## Methods

### Neural Network Model Details

We utilize a standard network model common in previous studies of the evolution of modularity [23, 57], extended with neuromodulatory neurons to add reinforcement learning dynamics [25, 69]. The network has five layers (Supp. S1 Fig) and is *feed-forward*, meaning each node receives inputs only from nodes in the previous layer and sends outputs only to nodes in the next layer. The number of neurons is 10/4/2 for the three hidden layers. The weights (connection strengths) and biases (activation thresholds) in the network take values in the range [-1, 1]. Following the paper that introduced the connection cost technique [23], networks are directly encoded [70, 71].

Information flows through the network from the input layer towards the output layer, with one layer per time step. The output of each node is a function of its inputs, as described in the next section.

### Learning Model

The neuromodulated ANN model in this paper was introduced by Soltoggio et al. [25], and adapted for the Sferes software package by Tonelli and Mouret [69]. It differs from standard ANN models by employing two types of neurons: *non-modulatory neurons*, which are regular, activity-propagating neurons, and *modulatory neurons*. Inputs into each neuron consist of two types of connections: *modulatory connections* *C*_*m*_ and *non-modulatory connections* *C*_*n*_ (normal neural network connections).

The output of a neuron is decided by the weighted sum of its non-modulatory input connections, as follows:

$$\begin{array}{c} a_{i}=\varphi \left(\sum_{j\in C_{n}}w_{ij}a_{j}+b_{i}\right) \end{array}$$ (1)

where *i* and *j* are neurons, *a*_*j*_ is the output of neuron *j*, *b*_*i*_ is the bias of neuron *i*, *w*_*ij*_ is the weight of the connection between neuron *i* and *j*, and *φ* is a sigmoid function that maps its input to a value in the range [−1, 1], allowing both positive and negative outputs.

Only *non-modulatory connections* (outgoing connections from non-modulatory neurons) are plastic. Their weight modification depends on the sum of modulatory inputs to the downstream neurons they connect to and a constant learning rate *η*. Their weight change is calculated by the following two equations:

$$\begin{array}{c} m_{i}=\varphi \left(\sum_{j\in C_{m}}w_{ij}a_{j}\right) \end{array}$$ (2)

$$\begin{array}{c} \forall j\in C_{n}:\Delta w_{ij}=\eta \cdot m_{i}\cdot a_{i}\cdot a_{j} \end{array}$$ (3)

Equation 2 describes how the modulatory input to each neuron is calculated. *φ* is a sigmoid function that maps its input to the interval [−1, 1] (thus allowing both positive and negative modulation). The sum includes weighted contributions from all modulatory connections.

Equation 3 describes how this modulatory input determines the learning rate of all incoming, non-modulatory connections to neuron *i*. *η* is a constant learning rate that is set to 0.04 in our experiments. The *a*_*i*_⋅*a*_*j*_ component is a regular Hebbian learning term that is high when the activity of the pre- and post-synaptic neurons of a connection are correlated [45]. The result is a Hebbian learning rule that is *regulated* by the inputs from neuromodulatory neurons, allowing the learning rate of specific connections to be increased or decreased in *specific circumstances*.

In control experiments without the potential for neuromodulation, all neurons were non-modulatory. Updates to the weights of their incoming connections were calculated via Equation 3 with *m*_*i*_ set to a constant value of 1.

### Evolutionary Algorithm

Our experiments feature a multi-objective evolutionary algorithm, which optimizes multiple objectives simultaneously. Specifically, it is a modification of the widely used Non-dominated Sorting Genetic Algorithm (NSGA-II) [72]. However, NSGA-II does not take into account that one objective may be more important than others. In our case, network *performance* is essential to survival, and minimizing the sum of connection costs is a secondary priority. To capture this difference, we follow [23] in having a *stochastic* version of Pareto dominance, in which the secondary objective (connection cost) only factors into selection for an individual with a given probability *p*. In the experiments reported here, the value of *p* was 0.75, but preliminary runs demonstrated that values of *p* of 0.25 and 0.5 led to qualitatively similar results, indicating that the results are robust to substantial changes to this value. However, a *p* value of 1 was found to overemphasize connection costs at the expense of performance, leading to pathological solutions that perform worse than the PA networks.

Evolutionary algorithms frequently get stuck in local optima [5] and, due to computational costs, are limited to small population sizes compared to biological evolution. To better capture the power of larger populations, which contain more diversity and thus are less likely to get trapped on local optima, we adopted the common technique of encouraging phenotypic diversity in the population [5, 73, 74]. Diversity was encouraged by adding a diversity objective to the multi-objective algorithm that selected for organisms whose network outputs were different than others in the population. As with performance, the diversity objective factors into selection 100% of the time (i.e. the probability *p* for PNSGA was 1). Technically, we register every choice (to eat or not) each individual makes and determine how different its sequence of choices is from the choices of other individuals: differences are calculated via a normalized bitwise XOR of the binary choice vectors of two individuals. For each individual, this difference is measured with regards to all other individuals, summed and normalized, resulting in a value between 0 and 1, which measures how different the behavior of this individual is from that of all other individuals. Preliminary experiments demonstrated that, for the problems in this paper, this diversity-promoting technique is necessary to reliably obtain functional networks in either treatment, and is thus a necessary prerequisite to conduct our study. This finding is in line with previous experiments that have showed that diversity is especially necessary for problems that involve learning, because learning problems are especially laden with local optima [74].

All experiments were implemented in the Sferes evolutionary algorithm software package [75]. The exact source code and experimental configuration files used in our experiments, along with data from all our experiments, are freely available in the online Dryad scientific archive at http://dx.doi.org/10.5061/dryad.s38n5.

### Mutational Effects

The variation necessary to drive evolution is supplied via random mutation. In each generation, every new offspring network is a copy of its parent that is randomly mutated. Mutations can add a connection, remove a connection, change the strength of connections, move connections and change the type of neurons (switching between modulatory and non-modulatory). Probabilities and details for each mutational event are given in Supp. S1 Table. We chose these evolutionary parameters, including keeping things simple by not adding crossover, to maintain similarity with related experiments on evolving modularity [23] and neuromodulated learning [76].

### Fitness Function

The fitness function simulates an organism learning associations in a world that fluctuates periodically between a summer and a winter season. During evolution, each individual is tested in four randomly generated environments (i.e. for four “lifetimes”, Fig. 2) that vary in which items are designated as food and poison, and in which *order* individuals encounter the items. Because there is variance in the difficulty of these random worlds, we test in 4 environments (lifetimes), instead of 1, to increase the sample size. We further increase the sample size to 80 environments (lifetimes) when measuring the performance of final, evolved, end-of-experiment individuals (e.g. Figs. 8 and 9). Individuals within the same generation are all subjected to the same four environments, but across generations the environments are randomized to select for learning, rather than genetically hard-coded solutions (Fig. 3). To start each environment (note: not season) from a clean slate, before being inserted in an environment the modulated weights of individuals are randomly initialized, which follows previous work with this neuromodulatory learning model [76]. Modulatory connections never change, and thus do not need to be altered between environments. In the runs without neuromodulation, all connections are reset to their genetically specified weights.

Throughout its life, an individual encounters different edible items several times (Fig. 2). Fitness is proportional to the number of food items consumed minus the number of poison items consumed across all environments (Supp. S7 Fig). Individuals that can successfully learn which items to eat and which to avoid are thus rewarded, and the best fitness scores are obtained by individuals that are able to retain this information across the fluctuating seasons (i.e. individuals that do not exhibit catastrophic forgetting).

### Modularity Calculations

Our modularity calculations follow those developed by Leicht and Newman for directed networks [67], which is an extension of the most well-established modularity optimization method [65]. That modularity optimization method relies on the maximization of a benefit function *Q*, which measures the difference between the number of connections within each module and the expected fraction of such connections given a “null model”, that is, a statistical model of random networks. High values of *Q* indicate an “unexpectedly modular” network.

For undirected networks, the null model traditionally corresponds to random networks constrained to have the same degree sequence as the network whose modularity is measured. Leicht and Newman extend this model to directed networks by distinguishing between the in-degree and out-degree of each node in the degree sequence [67]. The probability that the analyzed network has a connection between node *i* and *j* is therefore $k_{i}^{in}k_{j}^{out}/m$, where $k_{i}^{in}$ and $k_{j}^{out}$ are the in- and out-degrees of node *i* and *j*, respectively, *m* is the total number of edges in the network, and the modularity of a given decomposition for directed networks is as follows:

$$\begin{array}{c} Q=\frac{1}{m}\sum_{ij}\left[A_{ij}-\frac{k_{i}^{in}k_{j}^{out}}{m}\right]\delta_{ci,cj} \end{array}$$ (4)

*A*_*ij*_ is the connectivity matrix (1 if there is an edge from node *i* to node *j*, and 0 otherwise), *m* is the total number of edges in the network, and *δ*_*ci*, *cj*_ is a function that is 1 if *i* and *j* belong to the same module, and 0 otherwise. Our results are qualitatively unchanged when using layered, feed-forward networks as “null model” to compute and optimize *Q* (Supp. S2 Table).

Maximizing *Q* is an NP-hard problem [77], meaning it is necessary to rely on an approximate optimization algorithm instead of an exact one. Here we applied the *spectral optimization method*, which gives good results in practice at a low computational cost [67, 78]. As suggested by Leicht and Newman [67], each module is split in two until the next split stops increasing the modularity score.

### Experimental Parameters

Each experimental treatment was repeated 100 times with different stochastic events (accomplished by initiating experiments with a different numeric seed to a random number generator). Analyses are based on the highest-performing network from each trial. The experiments lasted 20,000 generations and had a population size of 400.

The environment had 2 different *seasons* (“summer” and “winter”). Each season lasted 5 *days*, and cycled through 3 *years* (Fig. 2). In each season, 2 *poisonous items* and 2 *nutritious items* were available, each item encoded by a separate input neuron (i.e. a “one-hot encoding” [64]).

Considering the fact that visiting objects in a different order may affect learning, the total number of possible different environments is 25,920. Each day we randomize the order in which food items are presented, yielding 4! = 24 different possibilities per day. There are in total 5 days per season, and an individual lives for 6 seasons, resulting in 5 × 6 = 30 days per lifetime (Fig. 2), and thus 24 × 30 = 720 different ways to visit the items in a single lifetime. In addition to randomizing the order items are visited in, the edibility associations agents are supposed to learn are randomized between environments. We randomly designate 2 of the 4 items as nutritious food, giving $\left(\begin{array}{c} 4\\ 2 \end{array}\right)=6$ different possibilities for summer and 6 different possibilities for winter. There are thus a total of 6 × 6 = 36 different ways to organize edibility associations across both seasons. In total, we have 720 × 36 = 25,920 unique environments, reflecting the 720 different ways food items can be presented and the 36 possible edibility associations.

As mentioned in the previous section, four of these environments were seen by each individual during evolution, and 80 of them were seen in the final performance tests. In both cases they were selected at random from the set of 25,920.

### Statistics

Unless otherwise stated, the test of statistical significance is the Mann-Whitney U test. 95% bootstrapped confidence intervals of the median are calculated by re-sampling the data 5,000 times. In Fig. 4, we smooth the plotted values with a median filter to remove sampling noise. The median filter has a window size of 11, and we plot each 10 generations, meaning the median spans a total of 110 generations.

### Measuring Learning and Retention

While measuring the forgetting and retention of evolved individuals (e.g. Figs. 8 and 9), further learning was disabled. The process is thus (1) learn food associations, (2) measure what was learned and forgotten without further learning, and (3) repeat. Disabling learning allows measurements of what has been learned without the evaluation changing that learned information.

## Supporting Information

S1 Fig The number and layout of the input, hidden, and output neurons. Inputs provide information about the environment. The output is interpreted as the decision to eat a food item or ignore it.
(TIFF)

S2 Fig The highest-performing networks from all of the 100 experiments in the PA treatment (part 1 of 2). Dark blue nodes are inputs that encode which type of food has been encountered. Light blue nodes indicate internal, non-modulatory neurons. Red nodes are reward or punishment inputs that indicate if a nutritious or poisonous item has been eaten. Orange nodes are neuromodulatory neurons that regulate learning. In the cases where an input neuron was modulatory, we indicate this with an orange circle around the neuron. In each panel, the left number reports performance and the right number reports modularity. We follow the convention from [23] of placing nodes in the way that minimizes the total connection length.
(TIFF)

S3 Fig The highest-performing networks from all of the 100 experiments in the PA treatment (part 2 of 2). See the previous figure caption for more details.
(TIFF)

S4 Fig The highest-performing networks from all of the 100 experiments in the P&CC treatment (part 1 of 2). Dark blue nodes are inputs that encode which type of food has been encountered. Light blue nodes indicate internal, non-modulatory neurons. Red nodes are reward or punishment inputs that indicate if a nutritious or poisonous item has been eaten. Orange nodes are neuromodulatory neurons that regulate learning. In the cases where an input neuron was modulatory, we indicate this with an orange circle around the neuron. In each panel, the left number reports performance and the right number reports modularity. We follow the convention from [23] of placing nodes in the way that minimizes the total connection length.
(TIFF)

S5 Fig The highest-performing networks from all of the 100 experiments in the P&CC treatment (part 2 of 2). See the previous figure caption for more details.
(TIFF)

S6 Fig Unnormalized values for Fig. 8 (left panel). Shows how old associations are forgotten as new ones are learned for the two experimental treatments. The treatment with a connection cost (P&CC) was able to learn the associations better and shows a more gradual forgetting in the first timesteps. Together, this leads it to outperform the regular treatment (PA) significantly when measuring how fast individuals forget. Note that networks were evolved with five days per season, so the results during those first five days are the most informative regarding the evolutionary mitigation of catastrophic forgetting: we show additional days to reveal longer-term consequences of the evolved architectures.
(TIFF)

S7 Fig The steps for evaluating the fitness of an individual. The example describes what happens when an agent encounters a food item during summer. For the winter season, the process is the same, but with winter inputs active instead of summer inputs.
(TIFF)

S1 Table The mutation operators along with their probabilities of affecting an individual. (TIFF)

S2 Table Two different null models for calculating the modularity score. The conventional way to calculate modularity is inherently relative: one computes the modularity of network N by searching for the modular decomposition (assigning N’s nodes to different modules) that maximizes the number of edges within the modules compared to the number of expected edges given by a statistical model of random, but similar, networks called the “null model”. There are different ways to model random networks, depending on the type of networks being measured and their topological constraints. Here, we calculated the modularity Q-score with two different null models, one modeling random, directed networks and the other modeling random, layered, feed-forward networks. When calculating modularity with either null model, P&CC networks are significantly more modular than PA networks. *A*_*ij*_ is 1 if there is an edge from node *i* to node *j*, and 0 otherwise, $k_{i}^{in}$ and $k_{j}^{out}$ are the in- and out-degrees of node *i* and *j*, respectively, *m* is the total number of edges in the network, *m*_*ij*_ is the number of edges between the layer containing node *i* and the layer containing node *j*, and *δ*_*ci*, *cj*_ is a function that is 1 if *i* and *j* belong to the same module, and 0 otherwise.
(TIFF)
