# Supplementary figures and images for: Neural Modularity Helps Organisms Evolve to Learn New Skills without Forgetting Old Skills
Source: PLoS Comput Biol. 2015 Apr 2;11(4):e1004128. doi: 10.1371/journal.pcbi.1004128 (PMC4383335; doi:10.1371/journal.pcbi.1004128)

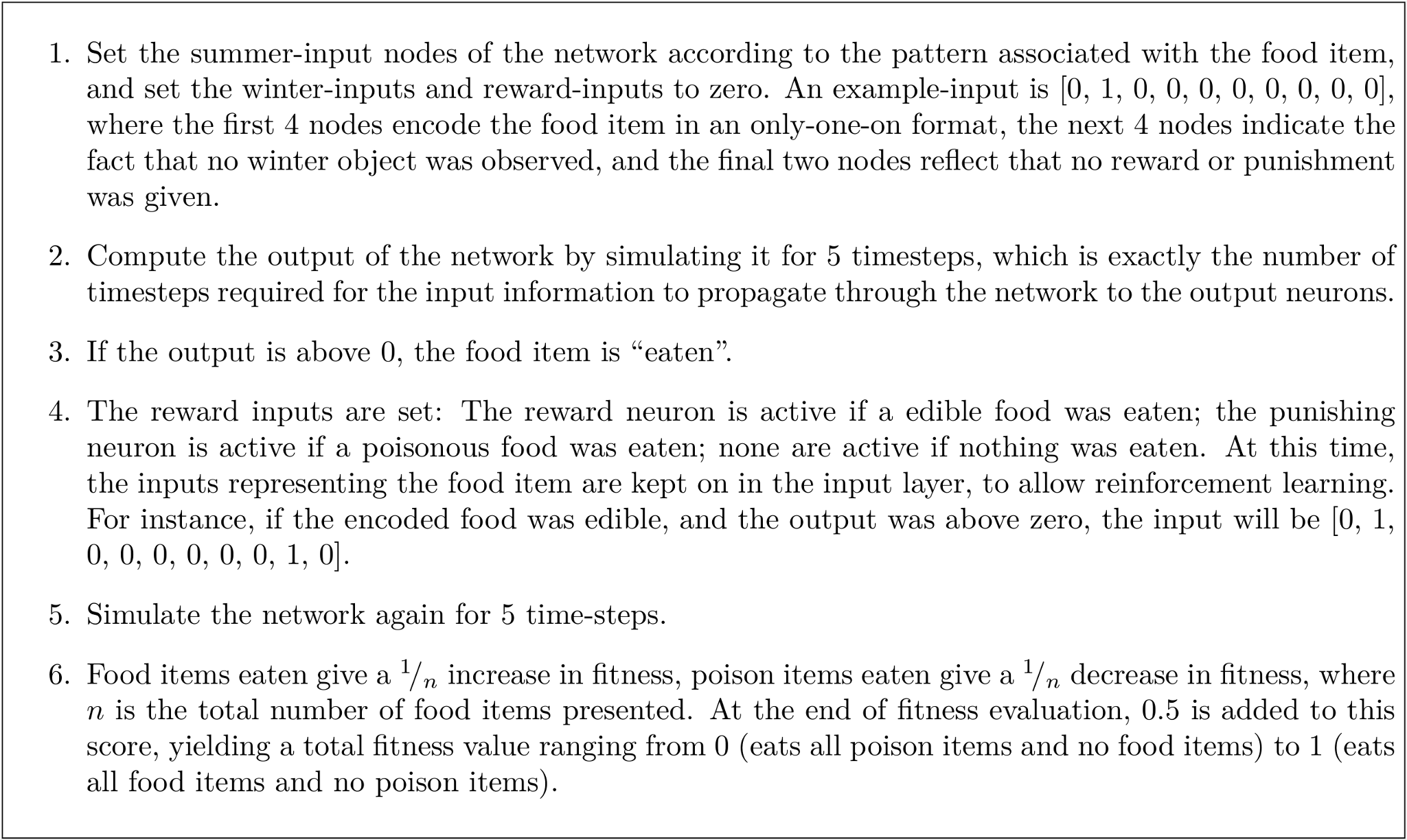

Supplement: S7 Fig — The example describes what happens when an agent encounters a food item during summer. For the winter season, the process is the same, but with winter inputs active instead of summer inputs. (TIFF) [file pcbi.1004128.s007.tiff]

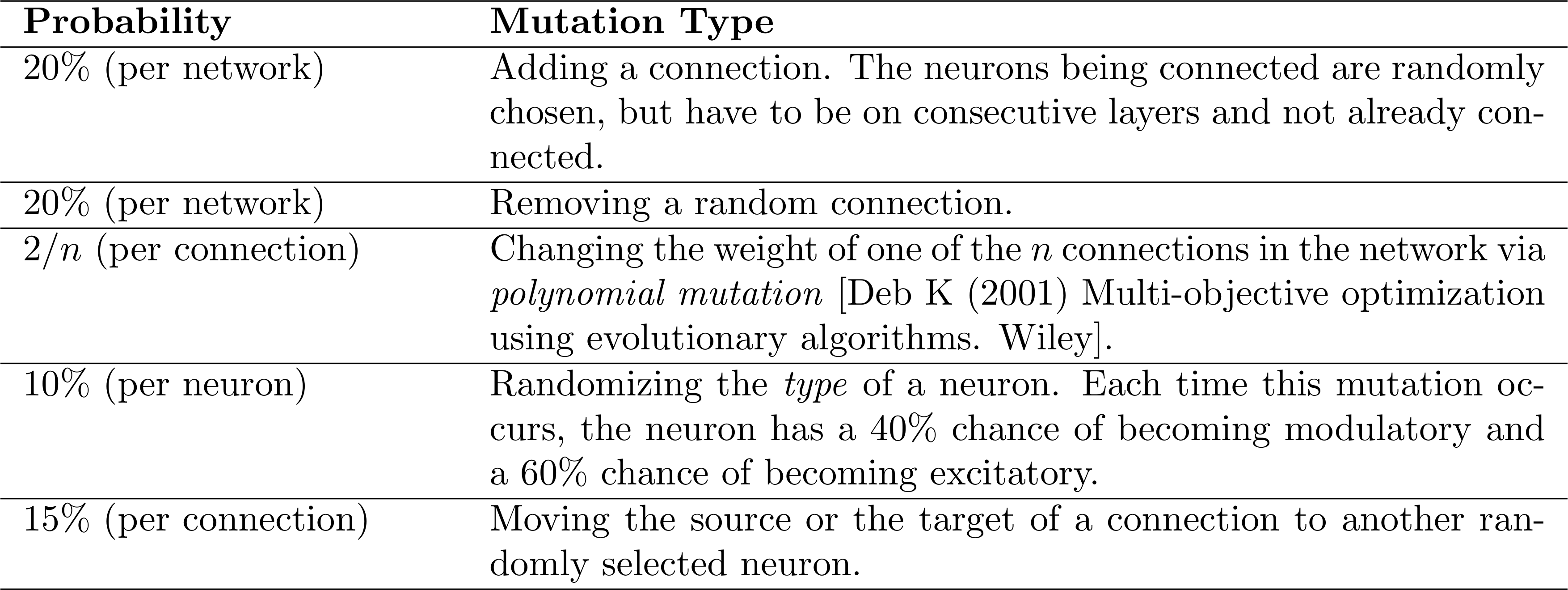

Supplement: S1 Table — (TIFF) [file pcbi.1004128.s008.tiff]

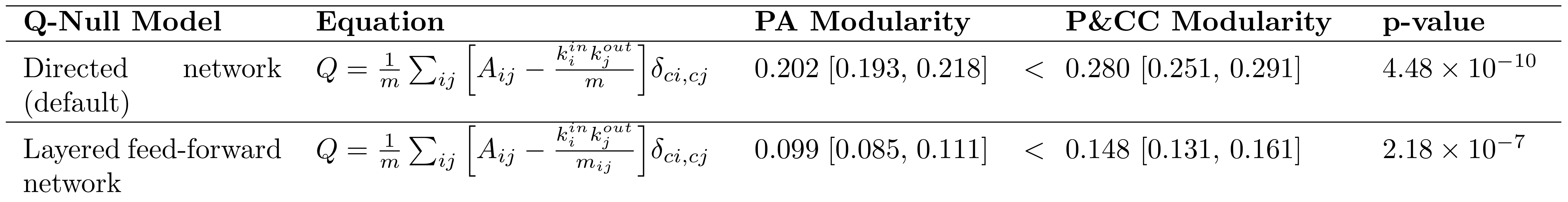

Supplement: S2 Table — The conventional way to calculate modularity is inherently relative: one computes the modularity of network N by searching for the modular decomposition (assigning N’s nodes to different modules) that maximizes the number of edges within the modules compared to the number of expected edges given by a statistical model of random, but similar, networks called the “null model”. There are different ways to model random networks, depending on the type of networks being measured and their topological constraints. Here, we calculated the modularity Q-score with two different null models, one modeling random, directed networks and the other modeling random, layered, feed-forward networks. When calculating modularity with either null model, P&CC networks are significantly more modular than PA networks. A ij is 1 if there is an edge from node i to node j, and 0 otherwise, kiin and kjout are the in- and out-degrees of node i and j, respectively, m is the total number of edges in the network, m ij is the number of edges between the layer containing node i and the layer containing node j, and δ ci, cj is a function that is 1 if i and j belong to the same module, and 0 otherwise. (TIFF) [file pcbi.1004128.s009.tiff]
